# Renal Tubular Acidosis Causing Acute Hypokalemic Paralysis in Systemic Lupus Erythematosus: Sjogren’s Syndrome Overlap

**DOI:** 10.7759/cureus.7555

**Published:** 2020-04-06

**Authors:** Mohammad Ammad Ud Din, Gaby Razzouk

**Affiliations:** 1 Internal Medicine, Rochester General Hospital, Rochester, USA

**Keywords:** acute hypokalemic paralysis, renal tubular acidosis, tubulointerstitial nephritis, lupus nephritis, sjogren's syndrome

## Abstract

Acute hypokalemic paralysis (AHP) is a reversible medical emergency either caused by excessive loss of potassium ions (K+) or increased intracellular shift of K+. Distal renal tubular acidosis (RTA) is an important differential to rule out in patients presenting with AHP. RTA is a constellation of disorders that have been associated with renal damage caused by autoimmune conditions such as systemic lupus erythematosus (SLE) and Sjögren's syndrome (SS). Here we present a case of a 44-year-old woman with a history of SLE in the absence of kidney disease who presented with AHP and was found to have distal RTA and antibodies positive for SS concerning tubulointerstitial nephritis in the setting of SS/SLE overlap syndrome.

## Introduction

Acute hypokalemic paralysis (AHP) is a potentially fatal but reversible medical condition caused by either excessive loss of potassium (K+) ions in urine in conditions like excessive diuretic use, Bartter’s syndrome, and renal tubular acidosis (RTA) or transcellular shifts of K+ seen in hypokalemic periodic paralysis (HPP) [1]. The two entities can be differentiated by the presence of excessive K+ in urine acidotic state ions in RTA, which is not seen with HPP [1]. RTA is a group of disorders involving a defect in the transport of bicarbonate and hydrogen ions across the cells lining the renal tubules, resulting in either decreased reabsorption of bicarbonate, or decreased excretion of hydrogen ions, or both [2]. Although renal involvement is well -established in autoimmune connective tissue diseases, AHP is rarely seen. Here, we present a case of a 44-year-old female with a history of systemic lupus erythematosus (SLE) who presented with hypokalemic paralysis and was found to have distal RTA in the setting of tubulointerstitial nephritis with antibodies positive for an SLE flare as well as Sjogren's syndrome (SS), concerning for SS/SLE overlap.

## Case presentation

A 44-year-old female with a past medical history of previously diagnosed systemic lupus erythematosus (SLE) presented after a fall because of a progressively worsening weakness of her lower extremities. She was also experiencing dry mouth, fatigue, numbness/tingling, along with morning stiffness in her hands for the past month. She was initially diagnosed with SLE three years ago when she presented with fatigue and a photosensitive facial rash and was prescribed hydroxychloroquine but she discontinued taking the medication after her symptoms abated and she was lost to follow up.

On presentation, she complained of generalized pain in her legs and vitals were stable. Her neurological exam was significant for the inability to move her legs against resistance or gravity (2/5 power as per the Medical Research Council scale of muscle strength). The sensation of crude touch and pain was preserved throughout.

She was found to have hypophosphatemia 1.1 mg/dl, severe hypokalemia of 1.6 mEq/L, and a hyperchloremic non-anion gap metabolic acidosis with venous pH of 7.21 in absence of any history of diarrhea, vomiting, alcohol abuse, or diuretic use, which was most consistent with type I or distal RTA (Table 1). Kidney function was normal, with a creatinine of 0.8 mg/dl and mild proteinuria. The thyroid profile and serum calcium were normal. The creatine kinase (CK) level was mildly elevated, likely secondary to her being immobile on the floor after the fall. The degree of acidosis and electrolyte imbalances was concerning for concurrent connective tissue disease and autoimmune workup revealed elevated titers of antibodies for antinuclear antibody (ANA), Sjogren's syndrome-related antigen A (SSA-A), double-stranded deoxyribonucleic (dsDNA), with normal complement (C3, C4) levels. Sjogren’s syndrome-related antigen B (SSA-B) antibody titer was also elevated, which was not elevated initially at the time of diagnosis (Table 2). She had also tested negative for antibodies targeting uridine rich U1 small nuclear riboprotein (u1-anti snRNP) previously.

**Table 1:** Summary of laboratory tests, including serum chemistry, venous blood gas, and thyroid profile Abbreviations: BUN: Blood urea nitrogen, Cr: creatinine, CK: creatine kinase, TSH: thyroid-stimulating hormone, PCO2: partial pressure, carbon dioxide, PO2: Partial pressure, oxygen Units: mEq/l: milliequivalents per liter, mg/dl: milligram per deciliter, ng/dl: nanogram per deciliter, mmHg: millimeter of mercury, uIU/ml: micro-international units per milliliter

Serum Chemistry	Result	Reference Range
Sodium	134	135-145
Potassium (mEq/l)	1.6	3.5-4.5
Chloride (mEq/l)	112	98-108
Bicarbonate (mEq/l)	10	22-30
BUN (mg/dl)	14	8-20
Creatinine (mg/dl)	0.8	0.7-1.2
Anion gap (mEq/l)	12	4-16
BUN/Cr	17.5	10-20
Phosphorus (mg/dl)	1.1	2.5-4.5
Magnesium (mg/dl)	2.4	1.4-2.5
Calcium (mg/dl)	9.2	8.5-10.4
CK (IU/l)	278	33-211
pH venous	7.21	7.32-7.42
PCO2, Venous (mmHg)	26	40-52
PO2, Venous (mmHg)	151	40-50
Bicarbonate, venous (mEq/l)	10	23-28
Base excess, venous (mEq/l)	-15.9	-2.0-3.0
TSH (uIU/ml)	1.06	0.55-4.78
Free T4 (ng/dl)	1.01	0.9-1.8

**Table 2:** Summary of laboratory tests for the autoimmune workup Abbreviations: ANA: antinuclear antibody, SSA: Sjogren’s syndrome-related antigen A, SSB: Sjogren’s syndrome-related antigen B, U1-snRNP: uridine rich U1 small nuclear riboprotein Units: mg/dl: milligram per deciliter, IU/l: international unit per liter, U/ml: unit per milliliter

Autoimmune Tests	Result	Reference Range
ANA screen	Positive	-
ANA titer	<640	<40
ANA pattern	Homogeneous	-
Anti-double stranded DNA (IU/l)	1234	0-29
Anti SSA antibody (U/ml)	445	0-100
Anti SSB antibody (U/ml)	54	0-19
Anti U1-snRNP antibody	Negative	-
C3 (mg/dl)	124	90-180
C4 (mg/dl)	21	18-45

The electrolyte imbalances were corrected with appropriate oral and intravenous supplements. Rheumatology was consulted for a possible lupus flare and a left kidney biopsy was performed, which showed mild to moderate interstitial nephritis with no evidence of immune complex deposition. The patient was restarted on hydroxychloroquine along with 20 mg/day prednisone. The interstitial nephritis was thought to be secondary to systemic lupus erythematous and possibly concurrent Sjogren’s disease. She was able to walk independently by the end of the second day of admission and was discharged on a prednisone taper with bicarbonate and potassium supplements to follow up with rheumatology and nephrology as an outpatient.

## Discussion

RTA is a group of disorders where the kidney fail to acidify urine [2]. AHP is the setting of distal RTA (dRTA) presents with increased urine K+ and non-anion gap metabolic acidosis, as was the case in our patient [1]. The result of the urinary K+ in a spot urine test (Table 3) should be interpreted with caution, keeping in mind that the serum K+ concentration was critically low. In cases where 24-hour urine K+ is not available, the urine K+ to urine creatinine ratio can help establish the diagnosis of RTA. A ratio of more than 2.5 mmol/L is highly suggestive of RTA as was the case in our patient [3]. RTA is rarely associated with SLE and when present, it usually is seen after significant tubulointerstitial damage [4]. Patients should be promptly started on treatment for lupus nephritis to prevent further progression of kidney disease [5]. Surprisingly, our patient had normal creatinine and mild proteinuria on presentation, which resolved on repeat testing during the follow-up visit. The biopsy results also only showed mild to moderate interstitial nephritis, for which she was started on 20 mg/day prednisone, which was increased to 40 mg/day as an outpatient the following week and later tapered over four weeks. Similarly, dRTA with hypokalemia to the extent that it causes AHP is not commonly seen.

**Table 3:** Urine spot test Units: mmol/l: millimole per liter, mmHg: millimeter of mercury

Spot Urine Test	Result
Osmolality	1.007
pH	6.5
Sodium (mmol/l)	33
Potassium (mmol/l)	11.2
Chloride (mmol/l)	37
Phosphorus (mmol/l)	<5.0
Creatinine (mmol/l)	3.6

A small review of six cases of RTA in the setting of SLE showed that two patients had proximal RTA while the other four had dRTA, all of which had mild hypokalemia with no cases of AHP [6]. However, cases of dRTA causing AHP and respiratory failure have been reported as the initial manifestation of SLE [7]. Interestingly, although our patient did not meet the criteria for mixed tissue connective disease (MCTD) because of the absence of antibodies against uridine rich U1 small nuclear riboprotein (U1 snRNP), she tested positive for SSA, and SSB antibodies suggestive of SLE-SS overlap syndrome [8]. Up to 30% of patients with SLE may have SS as well and a less aggressive course of lupus is this subset of patients as was the case in our patient till her presentation with AHP [9-10]. A study of 50 patients with SLE with concurrent antibodies for SS showed that SLE patients with both SSA and SSB positive antibodies had a skin manifestation as the most prominent feature while renal involvement was more common in patients with either SSA or SSB positive and anti-ds-DNA antibodies [11]. Unlike SLE, AHP with SS is well-documented [12-13]. SLE-SS overlap syndrome has been associated with life-threatening hypokalemia previously, which may indicate that dRTA is more often seen in these cases because of the concurrent SS in the setting of SLE [14]. The exact pathophysiology by which autoimmune conditions cause dRTA is not completely understood and is likely a multifactorial process. Studies have indicated that the reduced secretion of hydrogen ions (H+) in patients with SS maybe because of the downregulated expression of vacuolar H+ATPase in A-intercalated cells of the distal renal tubules [15]. Immunoglobulin G (IgG) autoantibodies against different protein targets involved in acid secretion cells have also been reported, however, it remains unclear whether the antibodies trigger the initial damage or are formed as a result of exposure of intracellular epitopes to B cells from the preceding cell damage [15].

## Conclusions

Autoimmune diseases like SLE can cause AHP secondary to dRTA even in the absence of the derangement of gross kidney function. The concurrent presence of antibodies for Sjogren's syndrome in SLE patients may increase the risk of AHP. Prompt treatment with potassium supplements and corticosteroids is recommended to prevent the progression of renal disease.
